# Prevalence of Abnormal Cardiovascular Magnetic Resonance Findings in Athletes Recovered from COVID-19 Infection: A Systematic Review and Meta-Analysis

**DOI:** 10.3390/jcm13113290

**Published:** 2024-06-03

**Authors:** Vasiliki Tsampasian, Emmanuel Androulakis, Ricardo Catumbela, Sabiha Gati, Michael Papadakis, Vassilios S. Vassiliou

**Affiliations:** 1 Norwich Medical School, Faculty of Medicine and Health Sciences, University of East Anglia, Norwich NR4 7UG, UK; 2 Norfolk and Norwich University Hospital, Norwich NR4 7TJ, UK; 3 Cardiovascular Clinical Academic Group, St. George’s University of London, London SW17 0RE, UK; e.androulakis@rbht.nhs.uk (E.A.); mipapada@sgul.ac.uk (M.P.); 4 Warrington and Halton Hospital NHS Trust, Warrington WA5 1QG, UK; 5 School of Medicine, Imperial College London, London SW7 2BX, UK; s.gati@rbht.nhs.uk; 6 Royal Brompton Hospital, London SW3 6NP, UK

**Keywords:** cardiovascular magnetic resonance, COVID-19, sports cardiology

## Abstract

**Background:** Competitive sports and high-level athletic training result in a constellation of changes in the myocardium that comprise the ‘athlete’s heart’. With the spread of the COVID-19 pandemic, there have been concerns whether elite athletes would be at higher risk of myocardial involvement after infection with the virus. This systematic review and meta-analysis evaluated the prevalence of abnormal cardiovascular magnetic resonance (CMR) findings in elite athletes recovered from COVID-19 infection. **Methods:** The PubMed, Cochrane and Web of Science databases were systematically search from inception to 15 November 2023. The primary endpoint was the prevalence of abnormal cardiovascular magnetic resonance findings, including the pathological presence of late gadolinium enhancement (LGE), abnormal T1 and T2 values and pericardial enhancement, in athletes who had recovered from COVID-19 infection. **Results:** Out of 3890 records, 18 studies with a total of 4446 athletes were included in the meta-analysis. The pooled prevalence of pathological LGE in athletes recovered from COVID-19 was 2.0% (95% CI 0.9% to 4.4%, *I*^2^ 90%). The prevalence of elevated T1 and T2 values was 1.2% (95% CI 0.4% to 3.6%, *I*^2^ 87%) and 1.2% (95% CI 0.4% to 3.7%, *I*^2^ 89%), respectively, and the pooled prevalence of pericardial involvement post COVID-19 infection was 1.1% (95% CI 0.5% to 2.5%, *I*^2^ 85%). The prevalence of all abnormal CMR findings was much higher among those who had a clinical indication of CMR. **Conclusions:** Among athletes who have recently recovered from COVID-19 infection, there is a low prevalence of abnormal CMR findings. However, the prevalence is much higher among athletes with symptoms and/or abnormal initial cardiac screening. Further studies and longer follow up are needed to evaluate the clinical relevance of these findings and to ascertain if they are associated with adverse events.

## 1. Introduction

High-level athletic training may provoke a spectrum of structural, functional and electrical myocardial adaptations that form the entity known as ‘athlete’s heart’ [1]. Exercise-induced cardiac remodelling or ‘athlete’s heart’ refers to the physiological myocardial remodelling that may occur as a result of the different pressure and volume loads on the heart muscle during chronic competitive training [2]. Cardiac magnetic resonance (CMR) is a valuable tool in the cardiovascular evaluation of elite athletes, as it allows accurate structural and functional cardiac assessment. It also enables the characterisation of the myocardial tissue, an important feature when differentiating the physiological adaptive features of athlete’s heart from pathological findings of cardiomyopathies.

There is evidence suggesting that non-specific myocardial fibrosis is more frequently encountered in athletes compared to sedentary individuals [3,4]. With the spread of the COVID-19 pandemic, concerns have been raised as to whether athletes would be at increased risk of myocardial involvement and subsequent adverse events. CMR is the gold standard technique that allows in-depth assessment of the myocardium and the diagnosis of pathological fibrosis and oedema [5]. Along with clinical evaluation and physical examination, CMR has an important role in the assessment of athletes before they return to intensive training and competitions [6]. Appropriate investigation is of paramount importance for a safe return to play for athletes across all sports disciplines [7].

Studies investigating the impact of COVID-19 infection on athletes’ myocardium have been conducted; however, there have been inconsistencies in the findings and observations between them, making interpretation and conclusions challenging. This systematic review and meta-analysis aim to critically evaluate and assess the prevalence of CMR-derived myocardial tissue characterisation abnormalities in athletes recovered from COVID-19 infection.

## 2. Materials and Methods

This systematic review and meta-analysis was conducted and reported according to the Preferred Reporting Items for Systematic Reviews and Meta-Analyses (PRISMA) guidelines [8] and has been registered with PROSPERO (registration number: CRD42023487503). The primary endpoint of this meta-analysis is the prevalence of abnormal cardiovascular magnetic resonance findings in athletes who have recovered from COVID-19 infection. These abnormal CMR-derived findings include the presence of a pathological myocardial pattern of late gadolinium enhancement (LGE) as well as abnormal T1 and T2 values. The primary findings of all the included studies are also discussed in the systematic review part.

### 2.1. Search Strategy

The PubMed, Cochrane and Web of Science databases were systematically searched from inception to 15 November 2023. The terms used for the search included ((COVID-19 OR Coronavirus) OR (severe acute respiratory syndrome coronavirus 2) OR (2019 ncov) OR (sars cov 2)) AND ((cardiovascular magnetic Resonance Imaging)) AND ((athletes) OR (sports)). The full search strategy is provided in the Supplementary Materials. After removing duplicates, two independent investigators (V.T., E.A.) performed title/abstract screening and subsequently full-text screening. Conflicts were resolved by discussion with a third investigator (V.S.V.), after which consensus was achieved. Two authors (V.T., E.A.) independently extracted data from the included studies using a standardised extraction form. Any disagreements were resolved by discussion with the senior author (V.S.V.). The data extracted included the study type, the number and characteristics of study participants, the number of participants who had CMR, the abnormal CMR findings and the time interval from infection to CMR.

### 2.2. Study Selection Process and Quality Assessment

All the studies that examined the prevalence of abnormal CMR findings in elite athletes ≥ 16 years old post COVID-19 infection were included in the meta-analysis. Only peer-reviewed articles were included and preprints were excluded. Studies published in languages other than English were also excluded. The PICO criteria for this systematic review and meta-analysis were as follows:

Population: Elite athletes ≥ 16 years old post COVID-19 infection.

Intervention: CMR assessment.

Comparison: None.

Outcome: Prevalence of abnormal CMR findings.

The Newcastle–Ottawa Scale, a nine-point measure assessing the quality of observational studies, was used to evaluate the studies included (Supplementary Table S2) [9].

### 2.3. Statistical Analysis

The event rates reported in each study for each of the outcomes investigated were used for the meta-analysis. A random-effects model was used to combine the event rates from all studies and calculate the pooled event rate expressed as a proportion. Statistical heterogeneity was assessed using *I^2^* statistics. Meta-regression analysis was performed to assess the study size effects. Publication bias was assessed using Egger’s test, the trim-and-fill method and funnel plots. Sensitivity analysis was performed where necessary in order to explore heterogeneity and to investigate the impact of potentially important clinical factors on the results (for example, analysis of studies that performed CMR only when clinically indicated, i.e., after abnormal initial screening or when participants had symptoms). In addition, sensitivity analysis was performed when small study effects tests were found to be significant in order to evaluate their impact on the overall results. Statistical analyses were conducted using the STATA 18 software (StataCorp. 2023. Stata Statistical Software: Release 18. College Station, TX, USA: StataCorp LLC). Statistical significance was defined as *p* < 0.05.

## 3. Results

The search of the PubMed, Cochrane and Web of Science databases yielded a total of 3890 records. After removal of duplicates, 3791 were screened at title/abstract level. After applying the inclusion and exclusion criteria, 86 studies underwent full text evaluation. Out of these, 18 studies with a total of 4446 athletes were included in the meta-analysis. The PRISMA flowchart for the study selection process is shown in Supplementary Figure S1. Table 1 summarises the study population and the characteristics of all the included studies.

There was variation between the studies in terms of the criteria by which athletes underwent a CMR evaluation. In five studies only, athletes with either symptoms or abnormal initial screening underwent a CMR scan [10,11,16,20,22]. In the rest of the studies, all athletes participating in the study had a CMR regardless of symptoms or initial screening.

In the majority of the studies, the athletes had their CMR evaluation within 4–6 weeks after their acute COVID-19 infection. However, in three studies, this time interval extended to a period of approximately 3 months [12,26,27]. The overall findings of each of the included studies are summarised in Table 2.

### 3.1. Prevalence of LGE

**Figure 1:**
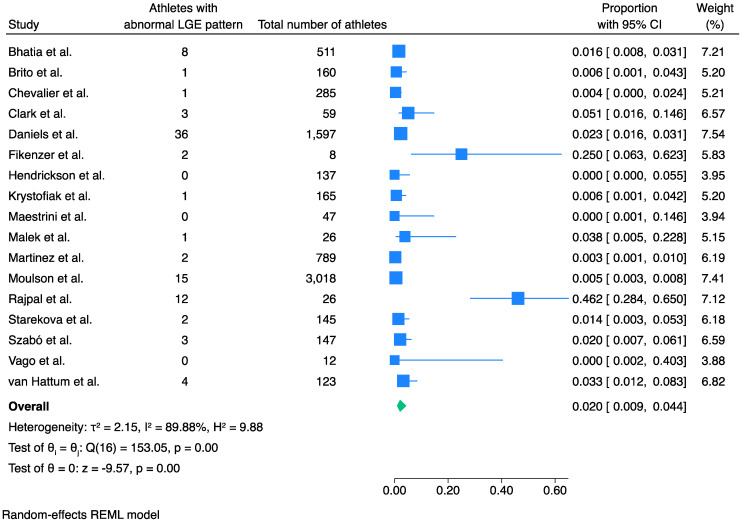
Pooled prevalence of pathological LGE pattern in athletes recovered from COVID-19 infection. Pathological LGE pattern was present in 2% of the athletes recovered from COVID-19 infection. Blue squares represent the prevalence of each study while the blue lines represent the 95% confidence intervals. The green shape represents the pooled prevalence of all studies. LGE, late gadolinium enhancement [10,11,12,13,14,15,16,17,18,19,20,21,22,23,24,25,26,27].

The pooled prevalence of pathological LGE in athletes recovered from COVID-19 was 2.0% (95% CI 0.9% to 4.4%, *I*^2^ 90%, *p* < 0.001) (Figure 1).

For this meta-analysis, the prevalence of a pathological LGE pattern was considered in relation to the total population. For this, we assumed that the asymptomatic athletes or those with normal initial screening would have normal CMR evaluation with no abnormal findings. Furthermore, unless otherwise stated by the authors, we considered insertion point fibrosis to be a normal variant in these elite athletes and hence this was not included as an abnormality.

Publication bias by Egger’s linear regress test was not significant (*p* = 0.16). Meta-regression analysis as per study size showed no significance (*p* = 0.27) (Supplementary Figure S2). Egger’s test for small study effects was not significant (*p* = 0.16) (Supplementary Figure S3) and trim-and-fill analysis showed no significant publication bias (Supplementary Figure S4). Two of the studies appeared to have a significantly higher prevalence of pathological LGE pattern compared to the other included studies [15,23]. In order to evaluate whether these studies have a major impact on the outcome, we have performed a sensitivity analysis without them. This confirmed the previously found statistically significant result and revealed a pooled prevalence of 1.3% (95% CI 0.8% to 2.2%, *I*^2^ 67%, *p* < 0.001) (Supplementary Figure S5).

We performed further sensitivity analyses in order to assess potential differences in LGE prevalence between studies with different patient selection criteria. Sensitivity analysis of the studies, in which only athletes with symptoms and/or abnormal initial screening were included, showed a higher prevalence of pathological LGE than expected at 7.8% (95% CI 2.2% to 24.1%, *I*^2^ 83%, *p* < 0.001) (Supplementary Figure S6). Sensitivity analysis of the studies in which CMR was performed on athletes regardless of symptoms or initial screening showed that the pooled prevalence of pathological LGE was 4.1% (95% CI 2.1% to 7.8%, *I*^2^ 86%, *p* < 0.001) (Supplementary Figure S7).

### 3.2. Prevalence of Abnormal T1 Values

**Figure 2:**
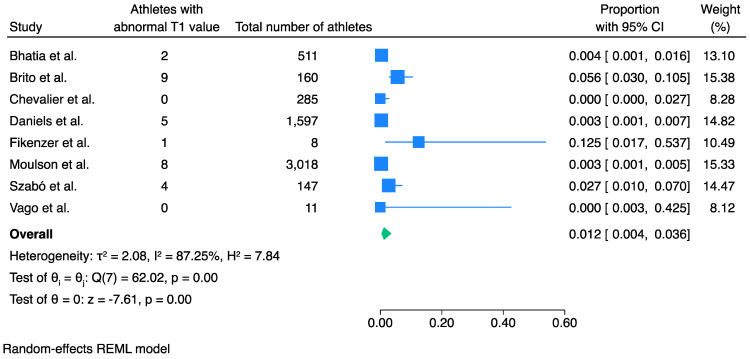
Pooled prevalence of abnormally elevated T1 values in athletes recovered from COVID-19 infection. An abnormal T1 value was found in 1.2% of the athletes recovered from COVID-19 infection. Blue squares represent the prevalence of each study while the blue lines represent the 95% confidence intervals. The green shape represents the pooled prevalence of all studies [10,11,12,14,15,21,25,26].

The prevalence of elevated T1 values in athletes recovered from COVID-19 infection was 1.2% (95% CI 0.4% to 3.6%, *I*^2^ 87%, *p* < 0.001) (Figure 2).

Egger’s test showed no significant small-study effects (*p* = 0.63) (Supplementary Figure S8), and trim-fill analysis showed no significant publication bias (Supplementary Figure S9). Meta-regression analysis as per study size showed that there was a statistically significant effect of the study size on the outcome (*p* = 0.02) (Supplementary Figure S10). Sensitivity analysis was therefore conducted excluding all the studies that had less than 50 participants, to elaborate whether size had any effect. This showed that the pooled prevalence was reduced to 0.8% (95% CI 0.3% to 2.5%, *I^2^* 80%, *p* < 0.001) (Supplementary Figure S11). This reduction, although significant, did not alter the clinical relevance. Furthermore, meta-regression analysis of this meta-analysis showed that the study size effect was now non-significant (*p* = 0.10), and Egger’s test demonstrated no significant publication bias (*p* = 0.21).

Sensitivity analysis with only the three studies that performed CMR when clinically indicated (only when symptoms were present and/or there was an abnormal initial screening) showed a higher prevalence of an abnormal T1 value of 11.5% (95% CI 5.5% to 22.5%, *I*^2^ 56%, *p* < 0.001) (Supplementary Figure S12).

### 3.3. Prevalence of Abnormal T2 Values

**Figure 3:**
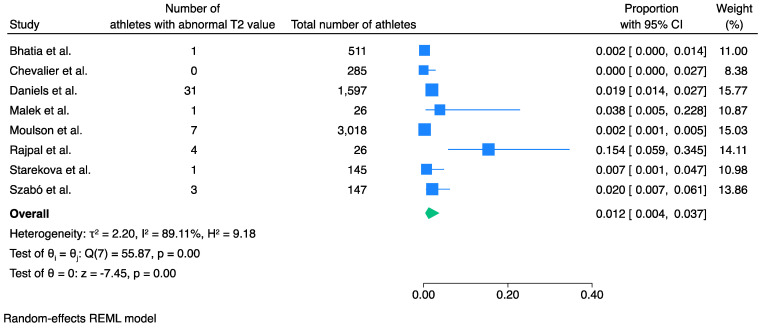
Pooled prevalence of abnormally elevated T2 values in athletes recovered from COVID-19 infection. An abnormal T2 value was found in 1.2% of the athletes recovered from COVID-19 infection. Blue squares represent the prevalence of each study while the blue lines represent the 95% confidence intervals. The green shape represents the pooled prevalence of all studies [10,12,14,19,21,23,24,25].

Meta-analysis of eight studies and 3,054 athletes showed that the pooled prevalence of elevated T2 values was 1.2% (95% CI 0.4% to 3.7%, *I*^2^ 89%, *p* < 0.001) (Figure 3).

Meta-regression analysis as per study size showed no significance (*p* = 0.68) (Supplementary Figure S13). Egger’s test showed no significant small-study effects (*p* = 0.13) (Supplementary Figure S14), and trim-fill analysis showed no significant publication bias (Supplementary Figure S15). Only two of the studies in this meta-analysis performed CMR when clinically indicated, with a total number of 136 athletes [10,21]; therefore, further sensitivity analysis was not performed.

### 3.4. Prevalence of Pericardial Involvement

**Figure 4:**
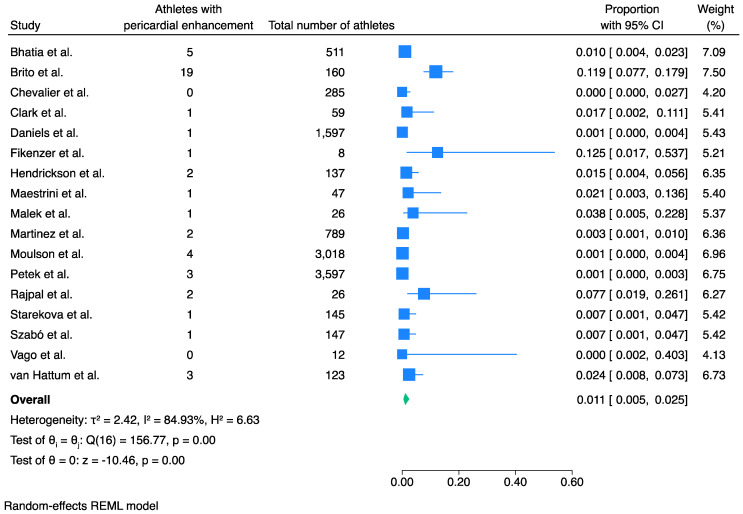
Pooled prevalence of pericardial enhancement in athletes recovered from COVID-19 infection. Pericardial involvement was present in 1.1% of the athletes recovered from COVID-19 infection. Blue squares represent the prevalence of each study while the blue lines represent the 95% confidence intervals. The green shape represents the pooled prevalence of all studies [10,11,12,13,14,15,16,18,19,20,21,22,23,24,25,26,27].

Meta-analysis of 17 studies and 4325 athletes showed that the pooled prevalence of pericardial involvement post COVID-19 infection was 1.1% (95% CI 0.5% to 2.5%, *I*^2^ 85%, *p* < 0.001) (Figure 4).

Meta-regression analysis for study size was significant (*p* = 0.001). Indeed, the bubble plot demonstrates an inverse linear correlation of the outcome with study size, with the smaller studies overestimating the prevalence of pericardial involvement (Supplementary Figure S16). A sensitivity analysis of only the six studies that included more than 200 athletes each [10,12,14,20,21,22] showed that the prevalence of pericardial involvement was less than 1% (Supplementary Figure S17). Egger’s test confirmed a significant small-study effect (*p* = 0.03) and visual assessment of the funnel plot did not show significant asymmetry (Supplementary Figures S18 and S19). This could support that the studies that had less stringent criteria in undertaking CMR and thus had more patients, had lower evidence of pericardial involvement. Therefore, the results should be interpreted with caution.

Sensitivity analysis of only the six studies that performed CMR when clinically indicated (only when symptoms were present and/or there was an abnormal initial screening) showed a prevalence of abnormal pericardial enhancement of 7.0% (95% CI 1.7% to 24.5%, *I*^2^ 90%, *p* < 0.001) (Supplementary Figure S20).

### 3.5. ECG Abnormalities and Elevated Troponin Levels in Athletes Post-COVID-19 Infection, in the Studies Reviewed in This Meta-Analysis

We also reviewed ECG and troponin levels in this cohort of patients. Ten studies showed that troponin was found to be elevated in a subset of athletes that had recently recovered from COVID-19 infection (Table 2). Due to the small number of studies and the differences in types of troponins tested (troponin I, troponin T, high sensitivity versus not high sensitivity or not specified), quantitative analysis was not deemed appropriate. The two higher observed percentages of athletes (15% and 14%) with elevated troponin are noted in the studies by Małek et al. and Daniels et al., respectively [14,19]. On both occasions, however, the high percentage reflects the small total number of participants that had their troponin tested (26 and 28, respectively) which, in turn, results in an overestimation of the observed outcome. Although elevated troponin was not necessarily associated with abnormal CMR findings, the opposite can be noted, as the majority of patients that exhibited pathological CMR findings also had elevated troponin levels [14,21,25].

The overall number of athletes with newly diagnosed ECG abnormalities after their recovery from acute COVID-19 infection was low across all the studies examined. Overall, only 64 out of a total of 2109 athletes (0.03%) from 17 studies were found to have ECG abnormalities, with the percentage in each study being 3.5% or less (Table 2).

## 4. Discussion

The results of this meta-analysis demonstrate that there is overall a low, but significant, prevalence of abnormal CMR findings in athletes that have recovered from acute COVID-19 infection. The prevalence of a pathological LGE pattern was 2.0% and the prevalence of elevated T1 and T2 values was 1.2% for each. In addition, pericardial involvement was prevalent in 1.1% of the athletes; however, this became <1% after the small studies were excluded from the analysis. Importantly, however, we found that the prevalence of abnormal findings was much higher when only participants with a clinical indication for CMR (i.e., ongoing symptoms or abnormal baseline tests such as ECG, echocardiography or blood biomarkers) were taken into consideration.

Our findings in elite athletes are in keeping with previous meta-analyses that demonstrated similar results. In a meta-analysis of 15 studies and 7988 athletes, Modica et al. demonstrated that the prevalence of COVID-19-related myocarditis among athletes was between 1% and 4% [28]. It was also shown that the prevalence of CMR abnormalities without necessarily meeting the Lake and Louise modified criteria, was 4% [28]. This is in agreement with our meta-analysis, in which we have examined the prevalence of all abnormal CMR findings, regardless of the diagnosis or not of myocarditis as per the Lake and Louise criteria. A further systematic review of 12 studies also demonstrated that athletes have an overall low risk of COVID-19-related myocardial and/or pericardial involvement and, subsequently, low risk of cardiac arrhythmias and cardiac death [29]. However, when we examined only the studies in which CMR was performed when clinically indicated, the prevalence of abnormal findings was significantly higher than the previously reported prevalence rates. This suggests the importance of CMR assessment in the appropriate patient population, i.e., those with a clinical indication, rather than non-selective use of CMR for all elite athletes recovered from COVID-19 infection.

The importance of pathological myocardial findings post COVID-19 infection stems from the fact that they may be associated with increased risk of malignant arrhythmias and sudden cardiac death [30]. Depending on the cardiomyopathic process, decisions on management and return-to-play (RTP) strategies are heavily based on the combination of symptomatology, clinical assessment and diagnostic evaluation. Management plans for athletes recently recovered from COVID-19 infection have been especially challenging as many of them exhibited mild or no symptoms. Nevertheless, the potential harmful impact of an underlying ongoing inflammatory process in the myocardium has prompted many physicians to utilise the strengths of CMR in order to assist decisions regarding the resumption of athletic activities.

Consensus statements suggest the use of CMR for athletes post COVID-19 infection with abnormal initial screening [5,31]. Our systematic review and meta-analysis provides a scientific rationale for the current guidelines. Abnormal CMR findings suggestive of myocardial injury were significantly more prevalent in athletes with an abnormal initial screening or clinical symptomatology. This suggests that the diagnostic yield of CMR for COVID-related cardiac involvement is much higher when it is clinically indicated. Conversely, screening with CMR is unlikely to add significantly to the athletes’ risk stratification post infection. The findings of our systematic review and meta-analysis, therefore, highlight how important CMR assessment is for athletes who are symptomatic during their COVID-19 infection or who have an abnormal initial screening before their return to competitive sports. At the same time, the presence of LGE in only 2% of the athletes is encouraging as it would indicate that the vast majority of individuals could return to sports soon, without any concerns about myocardial scarring.

In addition, our review was limited to the short-term period after acute COVID-19 infection. It is unknown if abnormal CMR findings persist for a prolonged period of time after the acute illness, but it is unlikely for LGE, representing scar tissue, to disappear in the longer-term. Crucially, though, the clinical relevance of such abnormal findings remains unknown. Further studies investigating the potential association between the presence of these findings and clinical outcomes in the longer term are needed in order to guide future management plans.

## 5. Limitations

Our review has limitations. Only studies published in English were included in this systematic review and meta-analysis. Although this is a source of selection bias, only three studies were excluded because of that reason; therefore, we do not feel that this has significantly affected the scope of the review. Furthermore, only elite athletes were included in this meta-analysis; therefore, further studies are needed to evaluate if these findings are observed in recreational athletes too. In addition, the majority of the studies included athletes from several types of sports without defining how many athletes from each sport were included. As such, it is impossible to make assumptions and associations between findings and a specific type of training (e.g., endurance, strength). Some of the meta-analyses performed had considerable statistical heterogeneity, which may have affected results. Although the vast majority of studies included had a time interval between diagnosis and CMR analysis of less than 6 weeks, in a couple of studies, this interval was approximately 3 months. This is another factor that may have contributed to the study heterogeneity. To try and account for this, we used both a random effects model and also undertook both meta-regression and sensitivity analysis. In addition, all the included studies were observational, which means that there is an unavoidable risk of bias. For this meta-analysis, we relied on pathological CMR patterns identified by the authors of each study included, and these results were not adjudicated in the same core lab. Finally, we accepted insertion point fibrosis as a normal variant, unless specifically reported as abnormal by the authors of the studies. This is because insertion point fibrosis in athletes is common and considered a physiological variant as it is not associated with an adverse prognosis [32].

## 6. Conclusions

Among all athletes who have recently recovered from COVID-19 infection, there is a low prevalence of abnormal CMR findings, including a pathological LGE pattern, abnormal T1 and T2 values and pericardial enhancement. However, among athletes with a clinical indication for CMR, the prevalence of abnormal CMR findings is higher—in the region of 4%. These findings suggest that the vast majority of athletes will not be at high risk of adverse events or outcomes following an acute COVID-19 infection. However, clinical evaluation of the athletes post COVID-19 infection, as well as initial cardiac screening, helps in risk stratification and identification of high-risk individuals. Clinicians should therefore consider CMR evaluation for athletes who are symptomatic during their COVID-19 infection and/or have an abnormal initial screening.

## Figures and Tables

**Table 1:** Summary and Characteristics of all the included studies.

Study	Study Design	Study Period	Study Population	Age	Tests Athletes Underwent	Number of Athletes That Had CMR	Time Interval from Infection/to CMR
Bhatia et al. [10]	Prospective observational study	March 2020–May 2022	511 soccer players (494 with no de novo ECG changes and 17 with de novo ECG changes)	21 years old (median)	Clinical assessment, ECG, echocardiogram, CMR	30 athletes underwent mandatory CMR, 17 athletes had CMR after abnormal ECG	15 days (median)
Brito et al. [11]	Cross-sectional observational study	June–August 2020	160 athletes from West Virginia University	19 years old (median)	Clinical assessment, questionnaire, ECG and echocardiogram (54), blood tests (Troponin, CRP, ESR, BNP) during individualised clinical assessments	48 athletes had CMR: symptomatic (mild or moderate illness) and asymptomatic with ECG/echocardiographic abnormalities)	27 days (median time interval from initial tests performed to the imaging assessment)
Chevalier et al. [12]	Prospective cohort study	June–December 2020	285 athletes (rugby players and student athletes)	25.8 years old (mean age of rugby players) and 20.1 years old (mean age of student athletes)	Clinical assessment, questionnaire, ECG and blood sampling (CRP, troponin I, D-Dimer, SARS-CoV-2 serology), Echocardiogram (including stress), Troponin, CMR	102 symptomatic and asymptomatic athletes that agreed to proceed with CMR assessment (CMR was offered to all)	51 ± 37 days
Clark et al. [13]	Retrospective case control study	March–December 2020	59 COVID-19-positive athletes, 60 athletic controls, and 27 healthy controls were included	20 years old (covid athletes) and 25 years old (athletic controls)	Clinical examination, ECG, troponin I, echocardiogram and CMR	The whole study population (symptomatic and asymptomatic subjects had CMR)	21.5 days (median)
Daniels et al. [14]	Retrospective observational study (Big Ten COVID-19 Registry from 13 Big Ten Universities in the USA across 17 sport disciplines)	March–December 2020	1597 athletes	Not provided	COVID-19-positive athletes underwent cardiac evaluation prior to CMR	All study participants had a CMR test (there were different diagnostic strategies across universities but, ultimately, only those who had CMR were included in the study)	22.5 days (median)
Fikenzer et al. [15]	Prospective cohort study	2020 (months not defined)	8 COVID-19-positive athletes and 4 non-infected athletes (controls)	27 years old (mean)	Clinical assessment, questionnaire, ECG, echo, CMR	All participants had CMR	19 ± 7 days
Hendrickson et al. [16]	Retrospective observational study	July–October 2020	137 collegiate athletes	20 years old (median)	Clinical assessment, ECG, Troponin	Anyone with abnormal test or clinical concern (*n* = 5)	16 days (median)
Krystofiak et al. [17]	Retrospective case series	August–December 2020	165 athletes	20 years old (median)	Trop, ecg, echo, CMR	All participants had CMR (regardless of symptoms)	25 days (median)
Maestrini et al. [18]	Prospective cohort study	November 2020	47 Italian Olympic athletes	26 years old (mean)	12 lead ECG, CPET, blood tests, 24-h ECG, spirometry, CMR	All participants had CMR (regardless of symptoms)	Median duration of the infection was 14 days, median time between the first negative covid test (NPS) and the RTP evaluation was 9 days
Małek et al. [19]	Retrospective cohort study	August–October 2020	26 Olympians	24 years old (median)	Clinical assessment, ECG, blood tests, CMR	All participants had CMR (regardless of symptoms)	32 days (median)
Martinez et al. [20]	Cross-sectional study	May–October 2020	789 professional athletes	25 years old (mean)	Clinical assessment, ECG, blood tests, echocardiogram	27 athletes with abnormal initial screening	19 days (mean)
Moulson et al. [21]	Prospective observational cohort study	September–December 2020	3018 athletes	20 years old (mean)	Clinical assessment, ECG, troponin, echocardiogram, CMR	317 athletes (primary screening with CMR performed in 198 athletes, but only 119 athletes had CMR as initial screening was abnormal)	33 days (median)
Petek et al. [22]	Prospective observational cohort study	September 2020–May 2021	3597 athletes with confirmed COVID and persistent (>3 weeks) or exertional symptoms	20 years old (mean)	Clinical assessment, ECG, troponin, echocardiogram, CMR	44 athletes with persistent symptoms, 137 with exertional symptoms	44 days (median)
Rajpal et al. [23]	Prospective cohort study	June–August 2020	26 athletes	19.5 years old (mean)	ECG, troponin, echocardiogram, CMR	All participants had CMR	CMR was performed after recommended quarantine (11–53 days)
Starekova et al. [24]	Retrospective observational study	January–November 2020	145 athletes	19.6 years old (mean)	Clinical assessment, ECG, troponin, echocardiogram, CMR	All participants had CMR	15 days (median)
Szabó et al. [25]	Observational case control study	July 2020–February 2021	147 athletes	23 years old (median)	Clinical assessment, questionnaire, ECG, troponin, echocardiogram, CMR	All participants had CMR [asymptomatic (*n* = 19) or with mild (*n* = 80), moderate (*n* = 43) or persistent (>4 weeks) (*n* = 5) symptoms]	32 days (median)
Vago et al. [26]	Prospective observational study	Not provided	12 athletes	23 years old (median)	Blood tests (CRP, NTproBNP, high sensitivity Troponin T), CMR	All participants had CMR	17 days for 10 female athletes, and 67 and 90 days in 2 male athletes, respectively.
Van Hattum et al. [27]	Prospective longitudinal study	May 2019–November 2022	123 COVID-19-positive athletes and 136 athletes (controls)	25 years old (mean)	Demographics, ECG, high sensitivity Troponin T, NTproBNP, CKMB, CMR	All participants had CMR (regardless of symptoms)	3.9 ± 2.9 months

CKMB, creatine kinase-myocardial band; CMR, cardiac magnetic resonance; CRP, C-reactive protein; ECG, electrocardiogram; NTproBNP, N-terminal pro b-type natriuretic peptide.

**Table 2:** Summary of findings of the studies included in the systematic review and meta-analysis.

Study	Number of Athletes with Elevated Troponin	Number of Athletes with One or More ECG Abnormalities	Number of Athletes That Had CMR	Number of Athletes with Pathological LGE Pattern	Number of Athletes with Abnormal T1 Values	Number of Athletes with Abnormal T2 Values	Number of Athletes with Pericardial Enhancement or Effusion
Bhatia et al. [10]	n/a	17 (3%)	47 (9.2%)	8 (17%)	2 (4%)	1 (2%)	5 (10%)
Brito et al. [11]	1 (3%)	1 (3%) (abnormal sinus tachycardia with ST segment and T wave changes)	48 (30%)	1 (2%)	9 (19%)	0	19 (39%)
Chevalier et al. [12]	8 (3%)	6 (2%)	102 (35.8%)	1 (1%)	0	0	0
Clark et al. [13]	0	0	59 (100%)	3 (5%)	Mild segmental increases in T1, T2, or extracellular volume were found in 39% of COVID-19-positive athletes, 13% of athletic controls, and 8% of healthy controls. Two asymptomatic COVID-19-positive athletes (3%) met criteria for myocarditis; one athlete had pericarditis. These athletes had normal electrocardiograms, troponin I, and echocardiograms with strain.		1 (2%)
Daniels et al. [14]	4 [14.3% of athletes with probable myocarditis (*n* = 28)]	1 [3.5% of athletes with probable myocarditis (*n* = 28)]	1597 (100%)	36 (2%)	5 (0.3%)	31 (2%)	1 0.1%)
Fikenzer et al. [15]	n/a	0	8 (100%)	2 (25%)	1 (12.5%)	0	1 (12.5%)
Hendrickson et al. [16]	4 (3%)	0	5 (3.6%)	0	0	0	2 (1.5%)
Krystofiak et al. [17]	0	0	165 (100%)	1 (0.6%)	1 (0.6%)	0	Not provided
Maestrini et al. [18]	1 (2.1%)	0 newly detected ECG abnormalities. 3 athletes had new PVCs during CPET.	47 (100%)	0	1 (2.1%)	1 (2.1%)	1 (2.1%)
Małek et al. [19]	4 (15%)	0	26 (100%)	1 (3.8%)	0	1 (3.8%)	1 (3.8%)
Martinez et al. [20]	6 (0.7%)	10 (1.3%)	27 (3.4%)	2 (0.25%)	Not provided	Not provided	2 (0.25%)
Moulson et al. [21]	24 (0.9%)	21 (0.7%)	317 (10.5%)	15 (4.7%)	8 (2.5%)	7 (2.2%)	4 (1.3%)
Petek et al. [22]	0	1 (0.8%)	181 (5%)	Five of forty-four (11.4%) athletes who underwent a CMR for exertional cardiopulmonary symptoms on return to exercise had probable or definite SARS-CoV-2 cardiac involvement, including 3 cases of pericardial involvement, 1 definite case of myopericardial involvement and 1 probable case of myopericardial involvement.			3 (6.8%)
Rajpal et al. [23]	0	0	26 (100%)	12 (46%)	0	4 (15%)	2 (7.7%)
Starekova et al. [24]	1 (0.7%)	1 (0.7%)	145 (100%)	2 (1.4%)	0	1 (0.7%)	1 (0.7%)
Szabó et al. [25]	6 (4.5%)	4 (2.7%)	147 (100%)	3 (2%)	4 (2.7%)	3 (2%)	1 (0.7%)
Vago et al. [26]	0	n/a	12 (100%)	0	0	0	0
Van Hattum et al. [27]	0	2 (1.6%)	123 (100%)	4 (3.3%)	0	0	3 (2.4%)

CPET, Cardio-pulmonary Exercise Test; ECG, Electrocardiogram; LGE, Late Gadolinium Enhancement; PVCs, Premature Ventricular Contractions.

## Data Availability

No new data were created in this study as data from already published studies were analysed. Data sharing is not applicable to this article.

## Supplementary Materials

The following supporting information can be downloaded at: https://www.mdpi.com/article/10.3390/jcm13113290/s1, Table S1. Search Strategy; Table S2. Newcastle-Ottawa Quality Assessment table for included cohort studies; Figure S1. PRISMA flow diagram of the study selection process; Figure S2. Meta-regression analysis of LGE prevalence as per study size; Figure S3. Funnel plot of LGE prevalence demonstrating no significant small-study effects; Figure S4. Funnel plot of LGE prevalence showing no significant publication bias; Figure S5. Sensitivity analysis for LGE prevalence; Figure S6. Sensitivity analysis for LGE prevalence including the studies in which CMR was performed when clinically indicated (presence of symptoms and/or abnormal initial screening; Figure S7. Sensitivity analysis for LGE prevalence of the studies in which CMR was done to athletes regardless of symptoms or initial screening; Figure S8. Funnel plot of abnormal T1 prevalence demonstrating no significant small-study effects; Figure S9. Funnel plot of abnormal T1 prevalence showing no significant publication bias; Figure S10. Meta-regression analysis of T1 prevalence as per study size; Figure S11. Sensitivity analysis for T1 prevalence excluding the studies with less than 50 participants; Figure S12. Sensitivity analysis for T1 prevalence including the studies in which CMR was performed when clinically indicated (presence of symptoms and/or abnormal initial screening; Figure S13. Meta-regression analysis of T2 prevalence as per study size; Figure S14. Funnel plot of abnormal T2 prevalence demonstrating no significant small-study effects; Figure S15. Funnel plot of abnormal T2 prevalence showing no significant publication bias; Figure S16. Meta-regression analysis of pericardial involvement as per study size; Figure S17. Sensitivity analysis for pericardial involvement including only large studies (with >200 participants); Figure S18. Funnel plot of pericardial involvement demonstrating significant small-study effects; Figure S19. Funnel plot of pericardial involvement showing no significant publication bias; Figure S20. Sensitivity analysis for pericardial enhancement including the studies in which CMR was performed when clinically indicated (presence of symptoms and/or abnormal initial screening.
