# Supplementary material for: Prevalence of Abnormal Cardiovascular Magnetic Resonance Findings in Athletes Recovered from COVID-19 Infection: A Systematic Review and Meta-Analysis
Source: J Clin Med. 2024 Jun 3;13(11):3290. doi: 10.3390/jcm13113290 (PMC11172781; doi:10.3390/jcm13113290)
Supplement: Supplementary file 1 [file jcm-13-03290-s001.zip › jcm-2973776-supplementary.pdf]

Supplementary Material

Prevalence of abnormal cardiovascular magnetic resonance findings in athletes recovered from COVID-19 infection

<u>**Tables**</u>

Table S1. Search Strategy
Table S2. Newcastle-Ottawa Quality Assessment table for included cohort studies

<u>**Figures**</u>

Figure S1. PRISMA flow diagram of the study selection process
Figure S2. Meta-regression analysis of LGE prevalence as per study size
Figure S3. Funnel plot of LGE prevalence demonstrating no significant small-study effects
Figure S4. Funnel plot of LGE prevalence showing no significant publication bias
Figure S5. Sensitivity analysis for LGE prevalence, excluding 2 studies with high prevalence LGE
Figure S6. Sensitivity analysis for LGE prevalence including the studies in which CMR was performed when clinically indicated (presence of symptoms and / or abnormal initial screening
Figure S7. Sensitivity analysis for LGE prevalence of the studies in which CMR was done to athletes regardless of symptoms or initial screening
Figure S8. Funnel plot of abnormal T1 prevalence demonstrating no significant small-study effects
Figure S9. Funnel plot of abnormal T1 prevalence showing no significant publication bias
Figure S10. Meta-regression analysis of T1 prevalence as per study size
Figure S11. Sensitivity analysis for T1 prevalence excluding the studies with less than 50 participants
Figure S12. Sensitivity analysis for T1 prevalence including the studies in which CMR was performed when clinically indicated (presence of symptoms and / or abnormal initial screening
Figure S13. Meta-regression analysis of T2 prevalence as per study size
Figure S14. Funnel plot of abnormal T2 prevalence demonstrating no significant small-study effects
Figure S15. Funnel plot of abnormal T2 prevalence showing no significant publication bias
Figure S16. Meta-regression analysis of pericardial involvement as per study size
Figure S17. Sensitivity analysis for pericardial involvement including only large studies (with >200 participants)
Figure S18. Funnel plot of pericardial involvement demonstrating significant small-study effects
Figure S19. Funnel plot of pericardial involvement showing no significant publication bias
Figure S20. Sensitivity analysis for pericardial enhancement including the studies in which CMR was performed when clinically indicated (presence of symptoms and / or abnormal initial screening

Table S1. Search Strategy

The total records identified consisted of the results of the following three search strategies:

A. Search Strategy 1
("covid 19"[All Fields] OR "covid 19"[MeSH Terms] OR "covid 19 vaccines"[All Fields] OR "covid 19 vaccines"[MeSH Terms] OR "covid 19 serotherapy"[All Fields] OR "covid 19 nucleic acid testing"[All Fields] OR "covid 19 nucleic acid testing"[MeSH Terms] OR "covid 19 serological testing"[All Fields] OR "covid 19 serological testing"[MeSH Terms] OR "covid 19 testing"[All Fields] OR "covid 19 testing"[MeSH Terms] OR "sars cov 2"[All Fields] OR "sars cov 2"[MeSH Terms] OR "severe acute respiratory syndrome coronavirus 2"[All Fields] OR "ncov"[All Fields] OR "2019 ncov"[All Fields] OR (("coronavirus"[MeSH Terms] OR "coronavirus"[All Fields] OR "cov"[All Fields]) AND 2019/11/01:3000/12/31[Date - Publication]) OR ("coronavirus"[MeSH Terms] OR "coronavirus"[All Fields] OR "coronaviruses"[All Fields]) OR ("sars cov 2"[MeSH Terms] OR "sars cov 2"[All Fields] OR "severe acute respiratory syndrome coronavirus 2"[All Fields]) OR ("sars cov 2"[MeSH Terms] OR "sars cov 2"[All Fields] OR "2019 ncov"[All Fields]) OR ("sars cov 2"[MeSH Terms] OR "sars cov 2"[All Fields] OR "sars cov 2"[All Fields])) AND (("cardiovascular system"[MeSH Terms] OR ("cardiovascular"[All Fields] AND "system"[All Fields]) OR "cardiovascular system"[All Fields] OR "cardiovascular"[All Fields] OR "cardiovasculars"[All Fields]) AND ("magnetic resonance imaging"[MeSH Terms] OR ("magnetic"[All Fields] AND "resonance"[All Fields] AND "imaging"[All Fields]) OR "magnetic resonance imaging"[All Fields])) AND ("athlete s"[All Fields] OR "athletes"[MeSH Terms] OR "athletes"[All Fields] OR "athlete"[All Fields] OR "athletically"[All Fields] OR "athlets"[All Fields] OR "sports"[MeSH Terms] OR "sports"[All Fields] OR "athletic"[All Fields] OR "athletics"[All Fields] OR ("sport s"[All Fields] OR "sports"[MeSH Terms] OR "sports"[All Fields] OR "sport"[All Fields] OR "sporting"[All Fields]))
B. Search Strategy 2
("covid 19"[All Fields] OR "covid 19"[MeSH Terms] OR "covid 19 vaccines"[All Fields] OR "covid 19 vaccines"[MeSH Terms] OR "covid 19 serotherapy"[All Fields] OR "covid 19 nucleic acid testing"[All Fields] OR "covid 19 nucleic acid testing"[MeSH Terms] OR "covid 19 serological testing"[All Fields] OR "covid 19 serological testing"[MeSH Terms] OR "covid 19 testing"[All Fields] OR "covid 19 testing"[MeSH Terms] OR "sars cov 2"[All Fields] OR "sars cov 2"[MeSH Terms] OR "severe acute respiratory syndrome coronavirus 2"[All Fields] OR "ncov"[All Fields] OR "2019 ncov"[All Fields] OR (("coronavirus"[MeSH Terms] OR "coronavirus"[All Fields] OR "cov"[All Fields]) AND 2019/11/01:3000/12/31[Date - Publication]) OR ("coronavirus"[MeSH Terms] OR "coronavirus"[All Fields] OR "coronaviruses"[All Fields]) OR ("sars cov 2"[MeSH Terms] OR "sars cov 2"[All Fields] OR "severe acute respiratory syndrome coronavirus 2"[All Fields]) OR ("sars cov 2"[MeSH Terms] OR "sars cov 2"[All Fields] OR "2019 ncov"[All Fields]) OR ("sars cov 2"[MeSH Terms] OR "sars cov 2"[All Fields] OR "sars cov 2"[All Fields])) AND (("cardiovascular system"[MeSH Terms] OR ("cardiovascular"[All Fields] AND "system"[All Fields]) OR "cardiovascular system"[All Fields] OR "cardiovascular"[All Fields] OR "cardiovasculars"[All Fields]) AND ("magnetic resonance imaging"[MeSH Terms] OR ("magnetic"[All Fields] AND "resonance"[All Fields] AND "imaging"[All Fields]) OR "magnetic resonance imaging"[All Fields]))

C. Search Strategy 3
("play and playthings"[MeSH Terms] OR ("play"[All Fields] AND "playthings"[All Fields]) OR "play and playthings"[All Fields] OR "play"[All Fields] OR ("sport s"[All Fields] OR "sports"[MeSH Terms] OR "sports"[All Fields] OR "sport"[All Fields] OR "sporting"[All Fields]) OR ("competition"[All Fields] OR "competitions"[All Fields] OR "competitive"[All Fields] OR "competitively"[All Fields] OR "competitiveness"[All Fields]) OR ("exercise"[MeSH Terms] OR "exercise"[All Fields] OR ("physical"[All Fields] AND "activity"[All Fields]) OR "physical activity"[All Fields]) OR ("exercise"[MeSH Terms] OR "exercise"[All Fields] OR "exercises"[All Fields] OR "exercise therapy"[MeSH Terms] OR ("exercise"[All Fields] AND "therapy"[All Fields]) OR "exercise therapy"[All Fields] OR "exercising"[All Fields] OR "exercise s"[All Fields] OR "exercised"[All Fields] OR "exerciser"[All Fields] OR "exercisers"[All Fields])) AND ("after"[All Fields] AND ("sars cov 2"[MeSH Terms] OR "sars cov 2"[All Fields] OR "covid"[All Fields] OR "covid 19"[MeSH Terms] OR "covid 19"[All Fields]))

Table S2. Newcastle-Ottawa Quality Assessment table for included studies

Study	Selection	Comparability	Outcome	Total	Quality of Study
Bhatia et al. [12]	***	*	***	7	High
Brito et al. [11]	***	*	**	6	Moderate
Chevalier et al. [13]	***	*	***	7	High
Clark et al. [14]	***	*	***	7	High
Daniels et al. [15]	***	*	***	7	High
Fikenzer et al. [16]	**	*	**	5	Moderate
Hendrickson et al. [10]	**	*	***	6	Moderate
Krystofiak et al. [17]	**	*	***	6	Moderate
Maestrini et al. [18]	**	*	***	6	Moderate
Małek et al. [19]	**	*	**	5	Moderate
Martinez et al. [9]	***	*	***	7	High
Moulson et al. [20]	***	*	***	7	High
Petek et al. [8]	***	*	***	7	High
Rajpal et al. [21]	**	*	***	6	Moderate
Starekova et al. [22]	***	*	***	7	High
Szabó et al. [23]	***	**	**	7	High
Vago et al. [24]	**	*	***	6	Moderate
Van Hattum et al. [25]	***	**	**	7	High

*Each star indicates a point for each component. Any study can obtain a maximum of four, two and three stars for each component (selection, comparability, outcomes) respectively. Trials with a total score of 7 or higher are considered to be high-quality studies, and those lower than 5 are considered as low-quality studies. These scores indicate at least moderate quality of the included trials.

Figure S1. PRISMA flow diagram of the study selection process

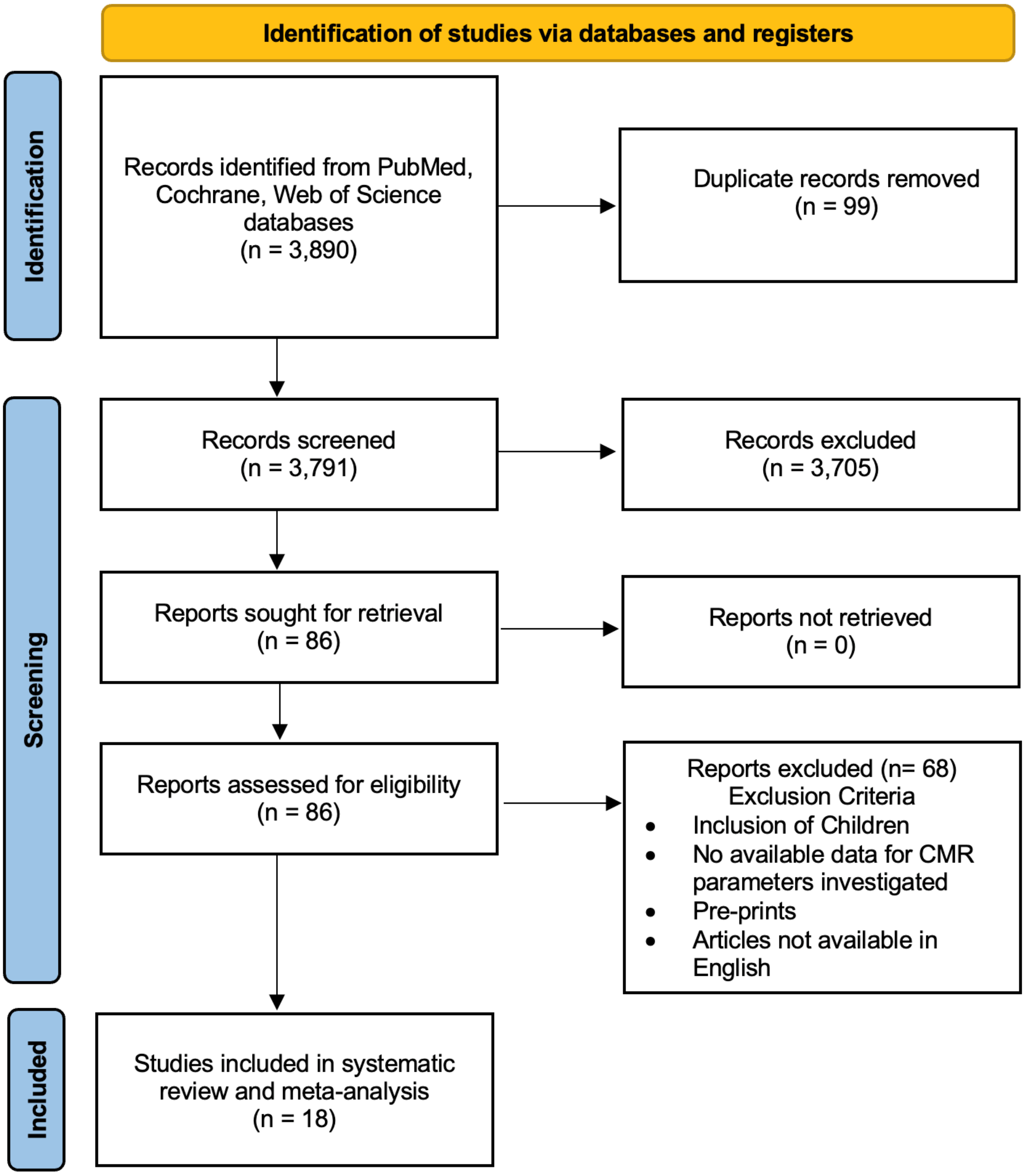

Figure S2. Meta-regression analysis of LGE prevalence as per study size

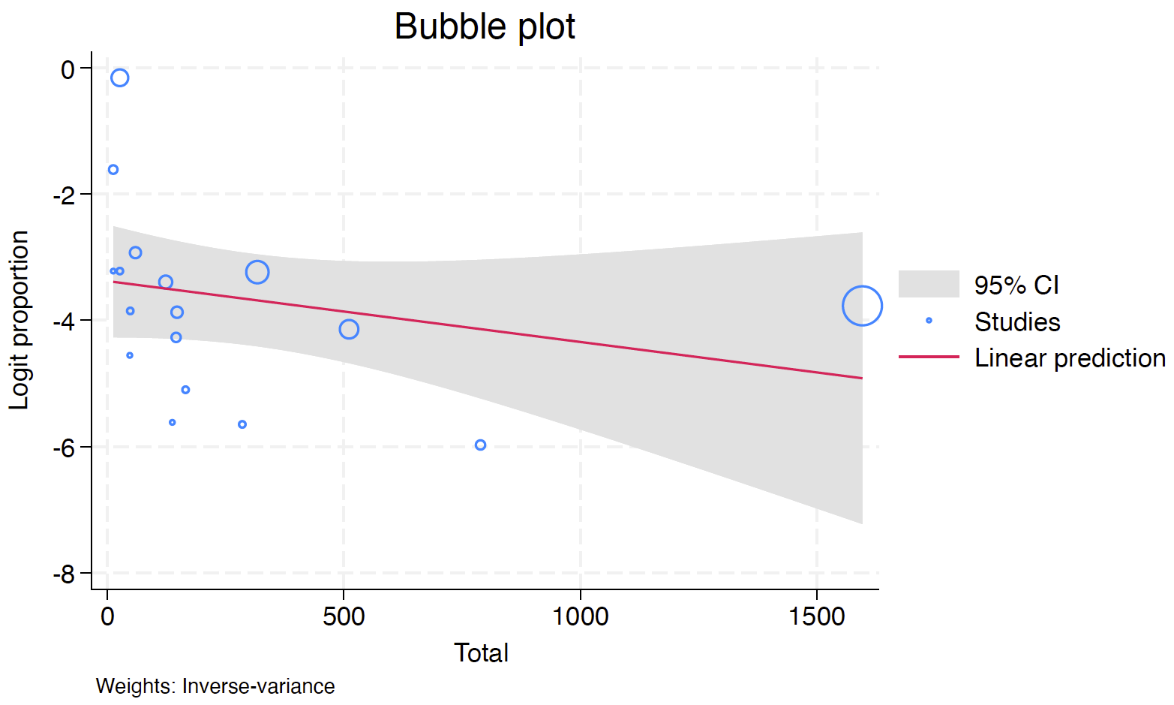

Figure S3. Funnel plot of LGE prevalence demonstrating no significant small-study effects

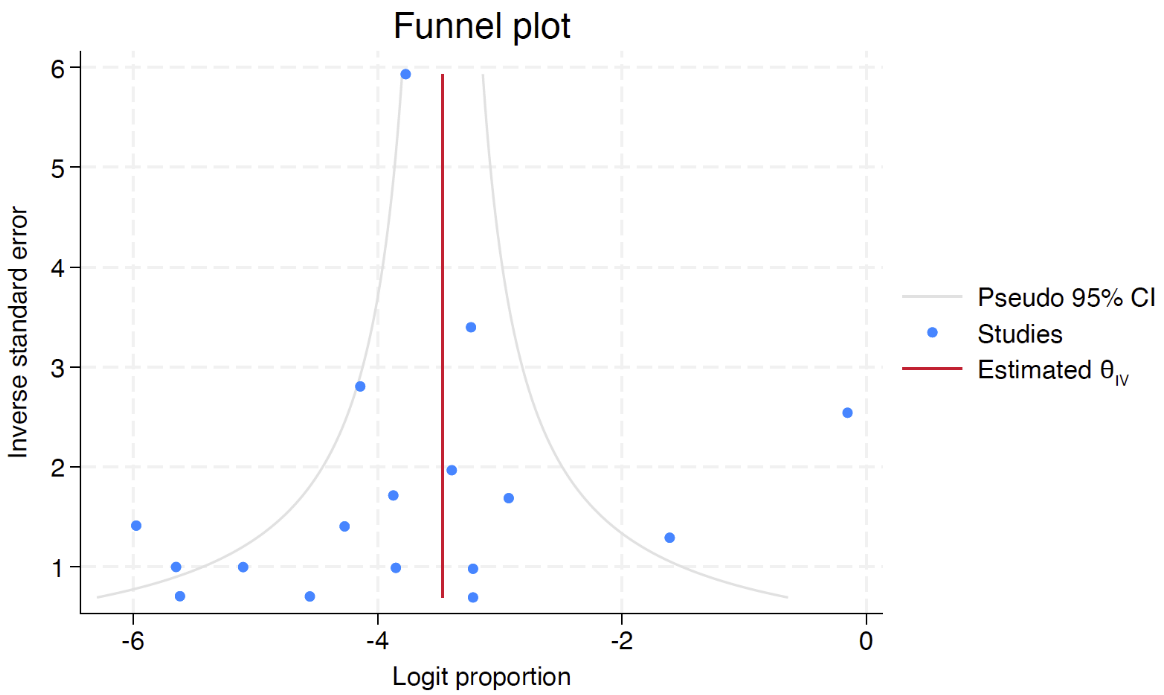

Figure S4. Funnel plot of LGE prevalence showing no significant publication bias

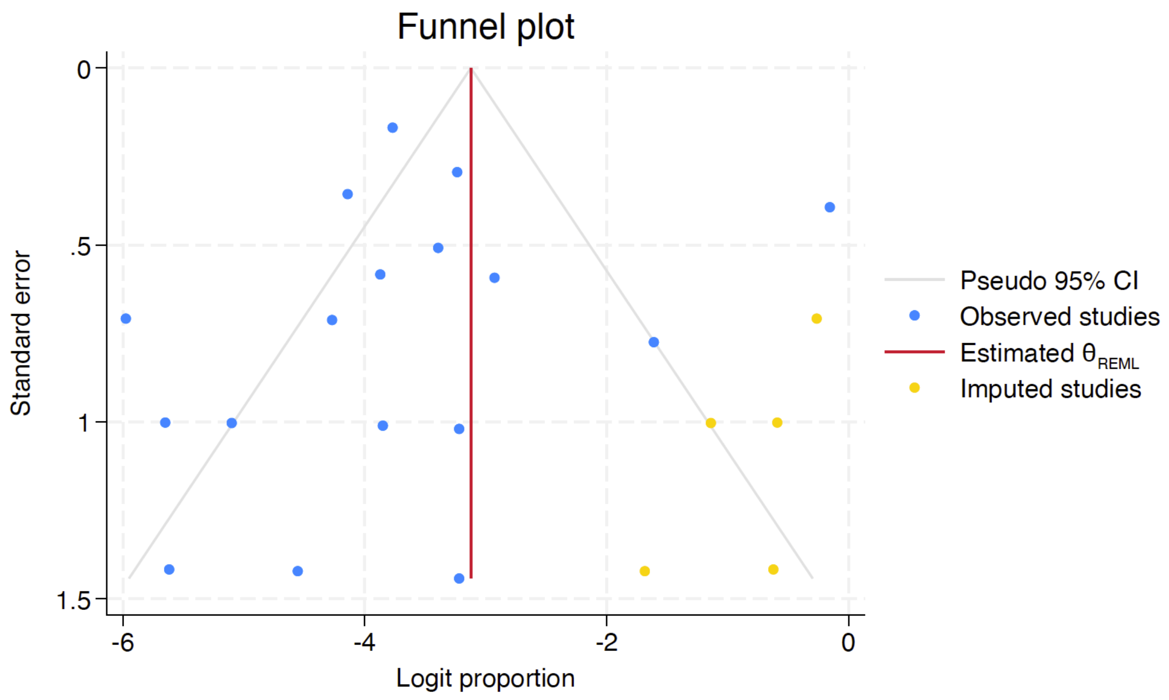

Figure S5. Sensitivity analysis for LGE prevalence, excluding 2 studies with high prevalence LGE

Study	Number of abnormal LGE pattern	Total number of athletes	Proportion with 95% CI	Weight (%)
Bhatia et al.	8	511	0.016 [0.008, 0.031]	10.80
Brito et al.	1	160	0.006 [0.001, 0.043]	4.51
Chevalier et al.	1	285	0.004 [0.000, 0.024]	4.52
Clark et al.	3	59	0.051 [0.016, 0.146]	7.97
Daniels et al.	36	1,597	0.023 [0.016, 0.031]	12.79
Hendrickson et al.	0	137	0.000 [0.000, 0.055]	2.71
Krystofiak et al.	1	165	0.006 [0.001, 0.042]	4.51
Maestrini et al.	0	47	0.000 [0.001, 0.146]	2.70
Malek et al.	1	26	0.038 [0.005, 0.228]	4.41
Martinez et al.	2	789	0.003 [0.001, 0.010]	6.78
Moulson et al.	15	3,018	0.005 [0.003, 0.008]	11.93
Starekova et al.	2	145	0.014 [0.003, 0.053]	6.74
Szabó et al.	3	147	0.020 [0.007, 0.061]	8.07
Vago et al.	0	12	0.000 [0.002, 0.403]	2.64
van Hattum et al.	4	123	0.033 [0.012, 0.083]	8.93
**Overall**			0.013 [0.008, 0.022]	

Heterogeneity: $\tau^2 = 0.50$, $I^2 = 67.12\%$, $H^2 = 3.04$

Test of $\theta_i = \theta_j$: $Q(14) = 44.43$, $p = 0.00$

Test of $\theta = 0$: $z = -16.55$, $p = 0.00$

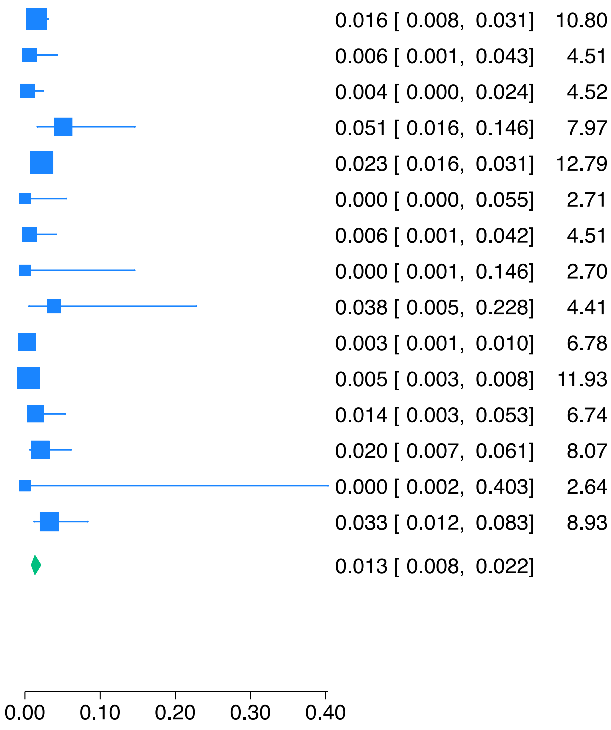

Random-effects REML model

Figure S6. Sensitivity analysis for LGE prevalence including the studies in which CMR was performed when clinically indicated (presence of symptoms and / or abnormal initial screening

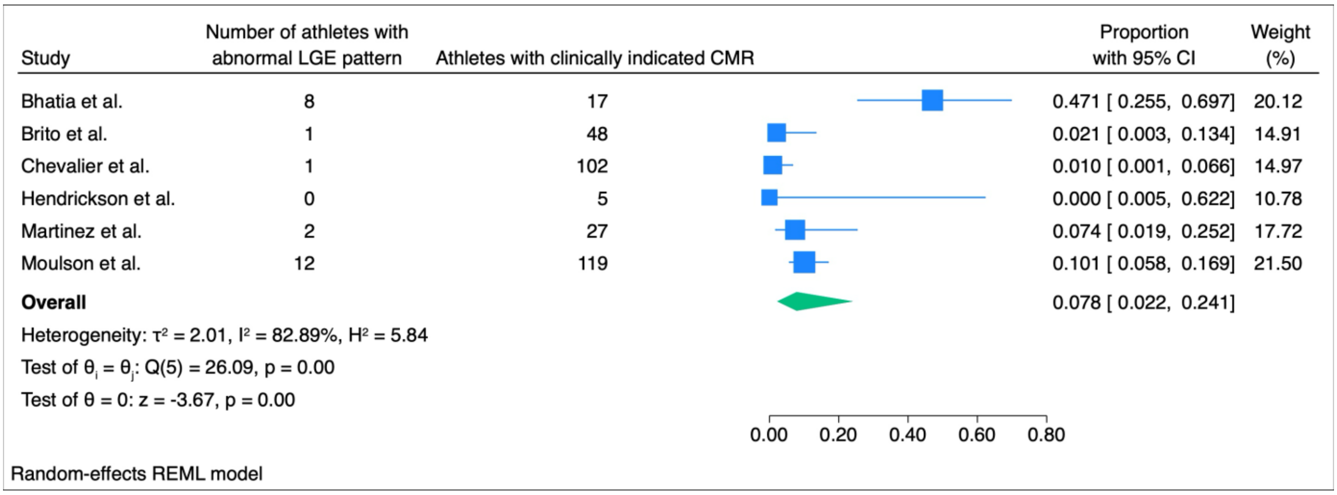

Figure S7. Sensitivity analysis for LGE prevalence of the studies in which CMR was done to athletes regardless of symptoms or initial screening

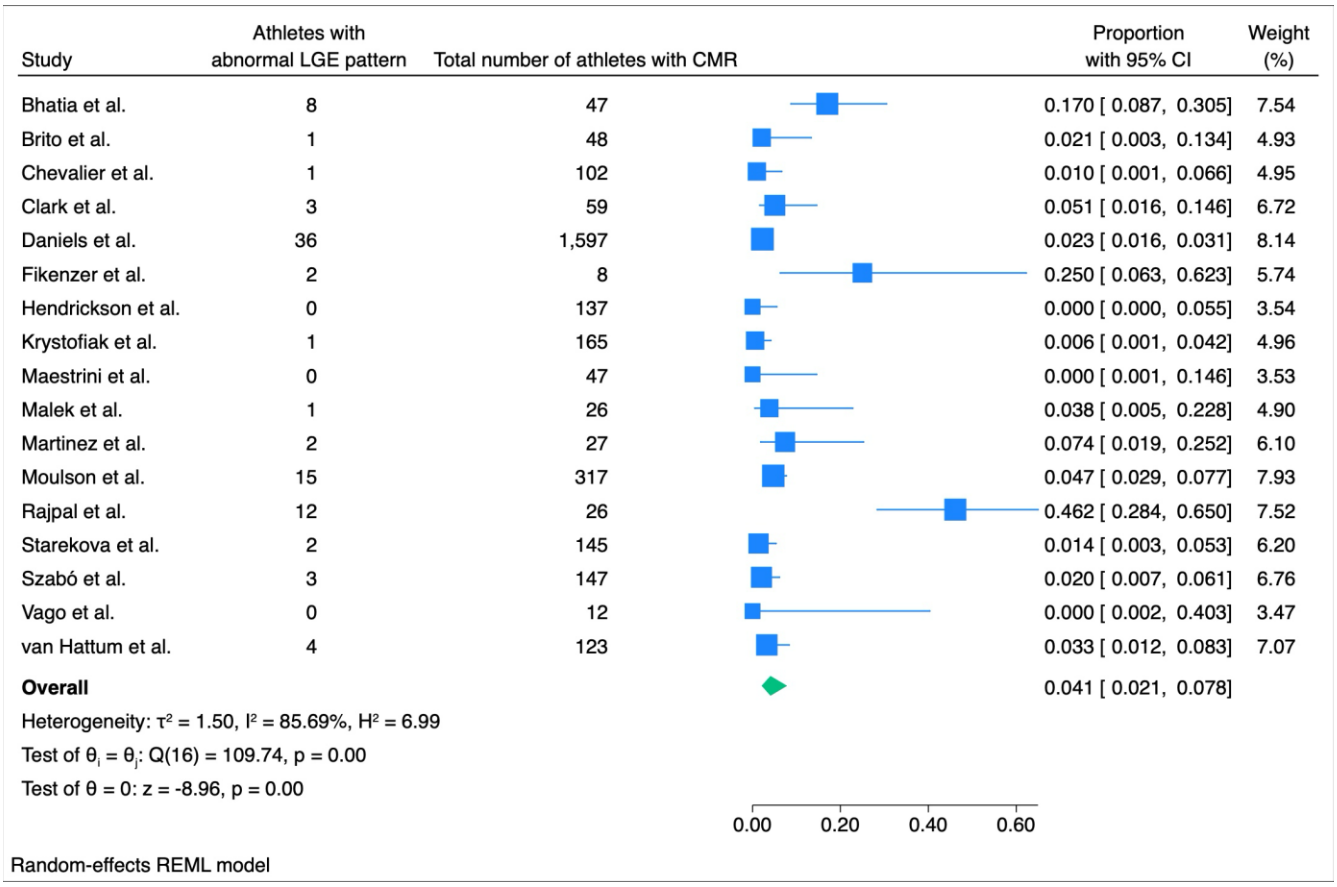

Figure S8. Funnel plot of abnormal T1 prevalence demonstrating no significant small-study effects

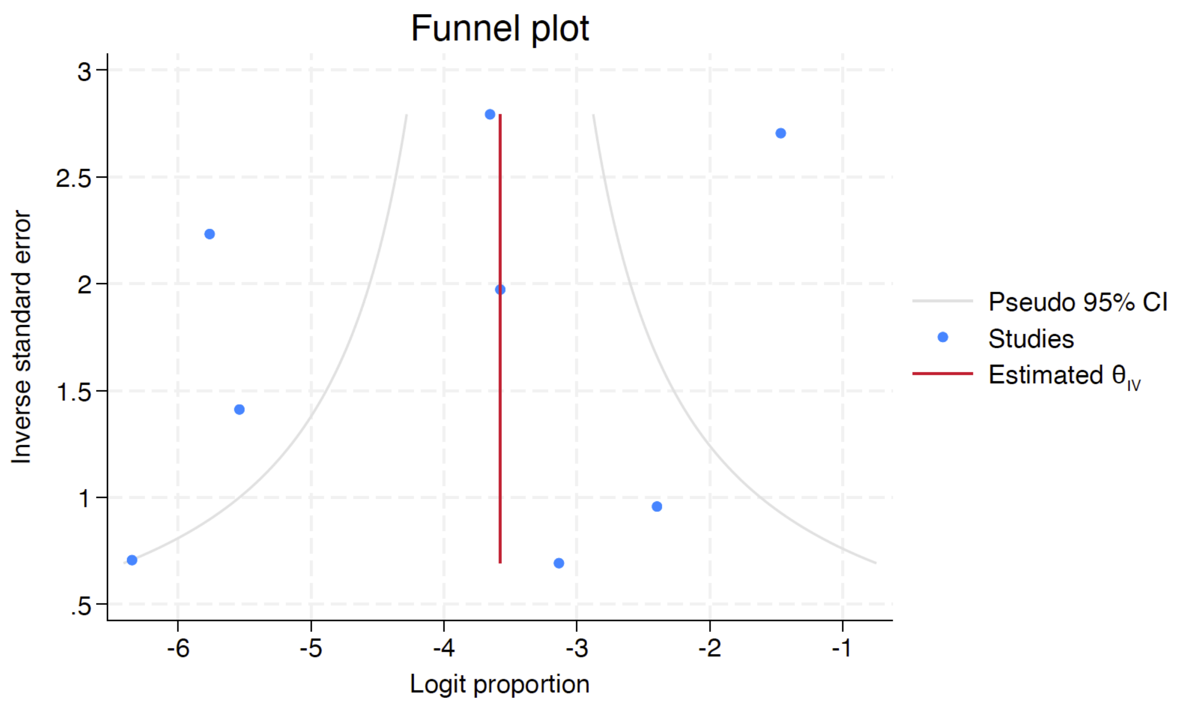

Figure S9. Funnel plot of abnormal T1 prevalence showing no significant publication bias

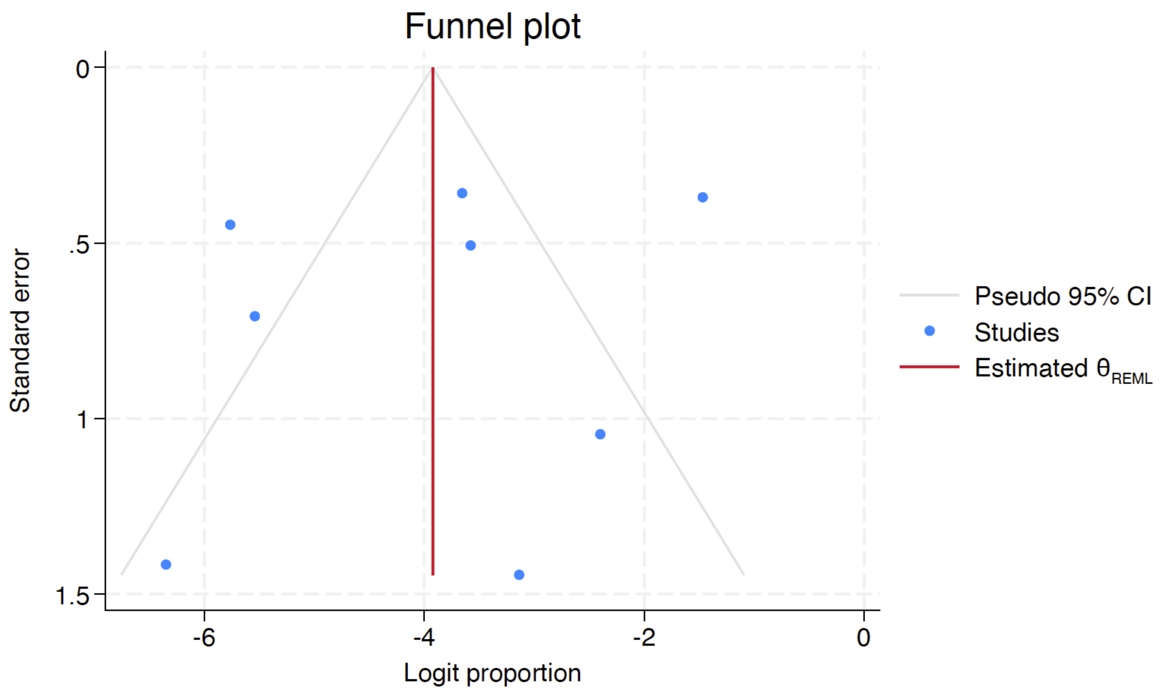

Figure S10. Meta-regression analysis of T1 prevalence as per study size

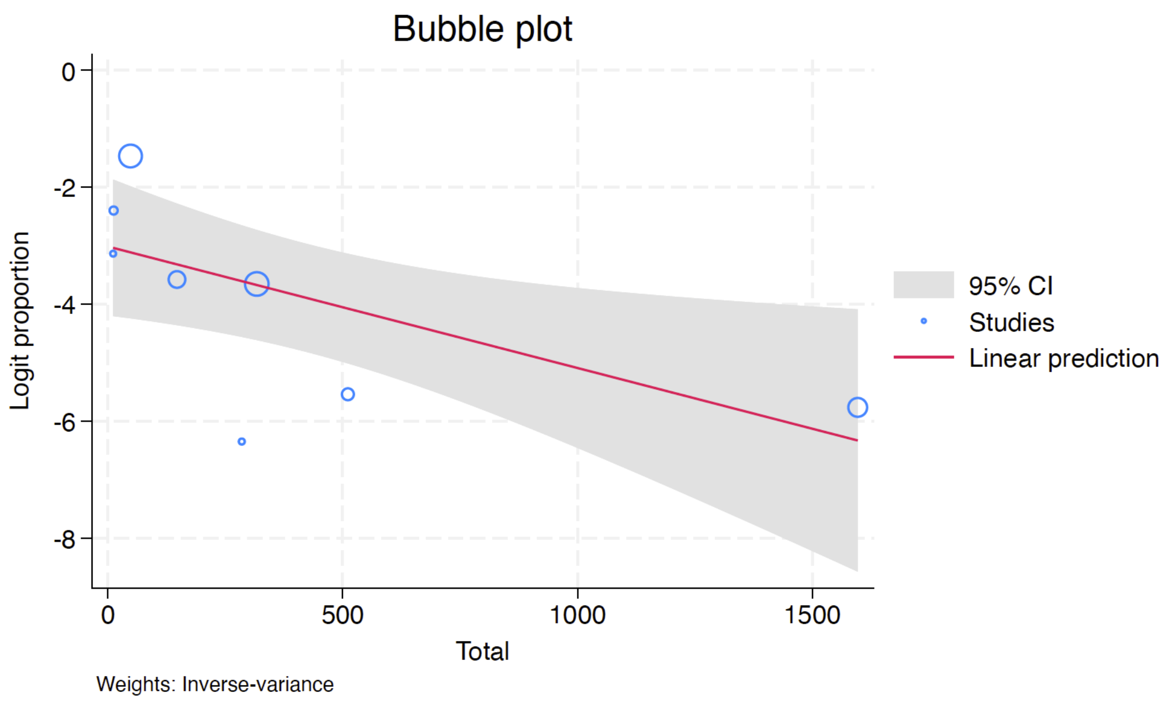

Figure S11. Sensitivity analysis for T1 prevalence excluding the studies with less than 50 participants

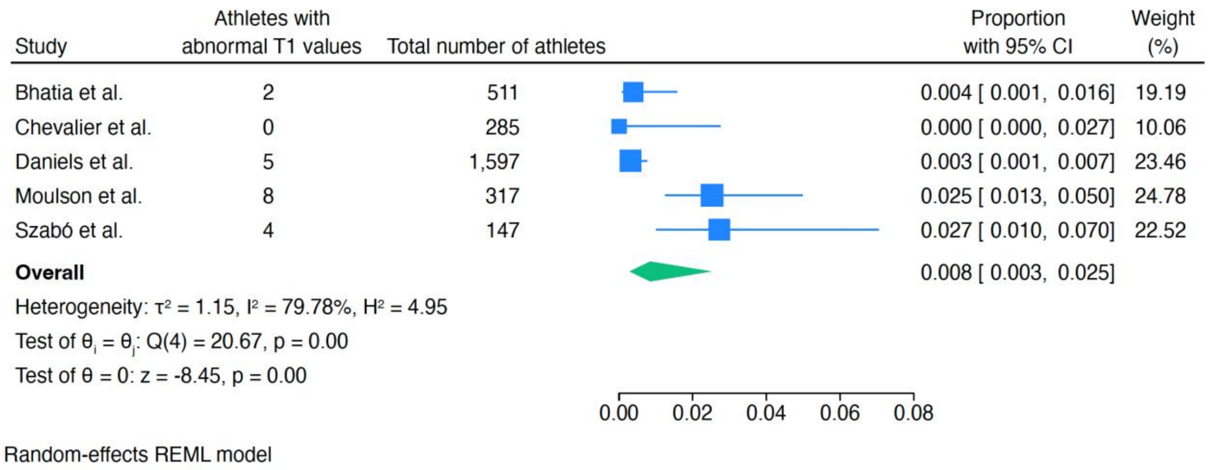

Figure S12. Sensitivity analysis for T1 prevalence including the studies in which CMR was performed when clinically indicated (presence of symptoms and / or abnormal initial screening

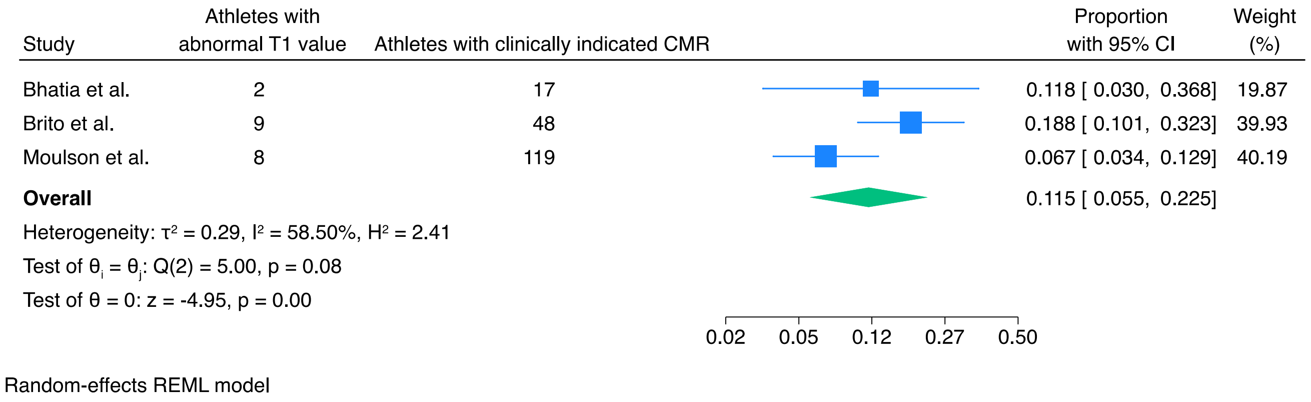

Figure S13. Meta-regression analysis of T2 prevalence as per study size

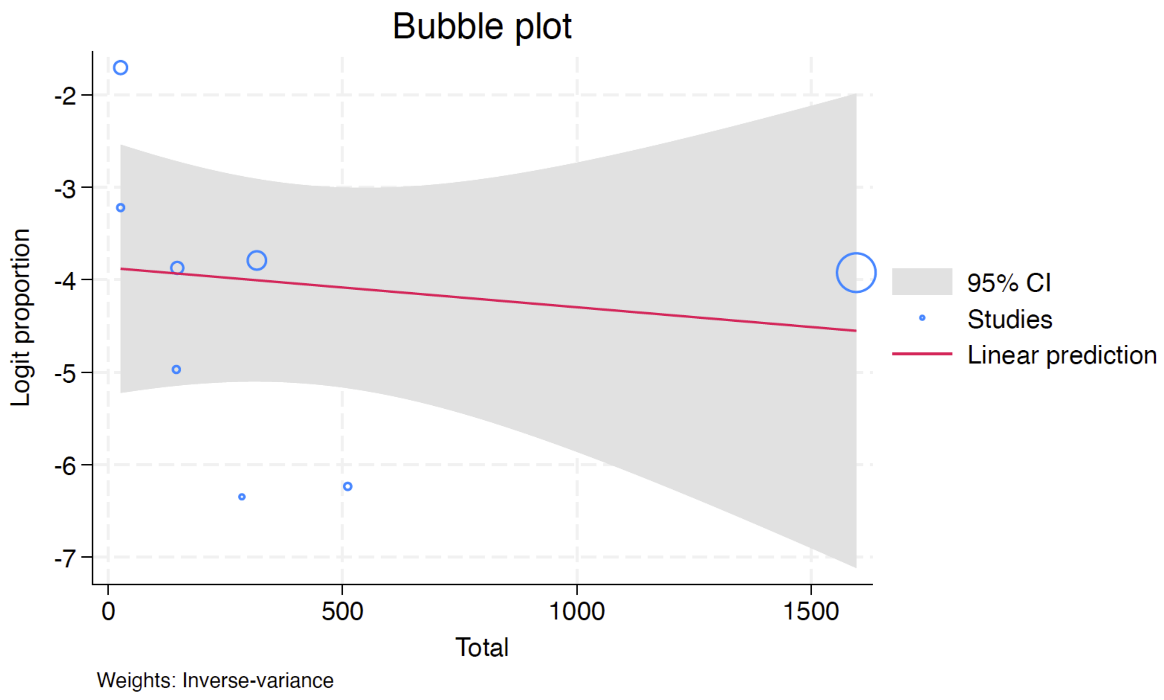

Figure S14. Funnel plot of abnormal T2 prevalence demonstrating no significant small-study effects

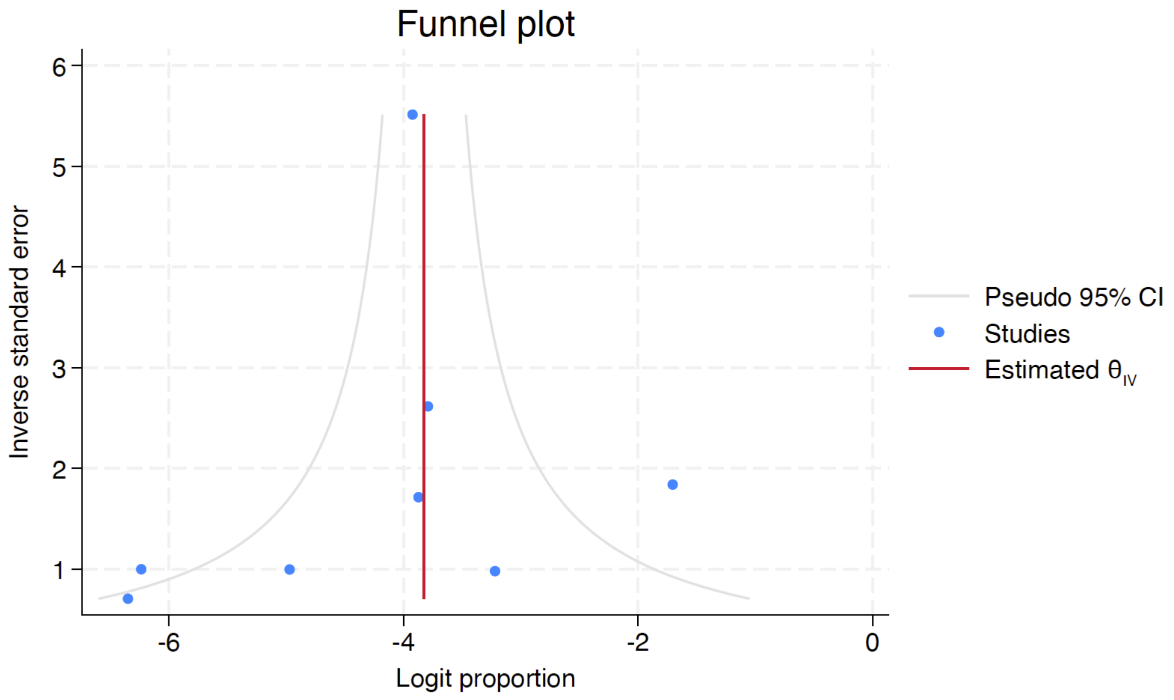

Figure S15. Funnel plot of abnormal T2 prevalence showing no significant publication bias

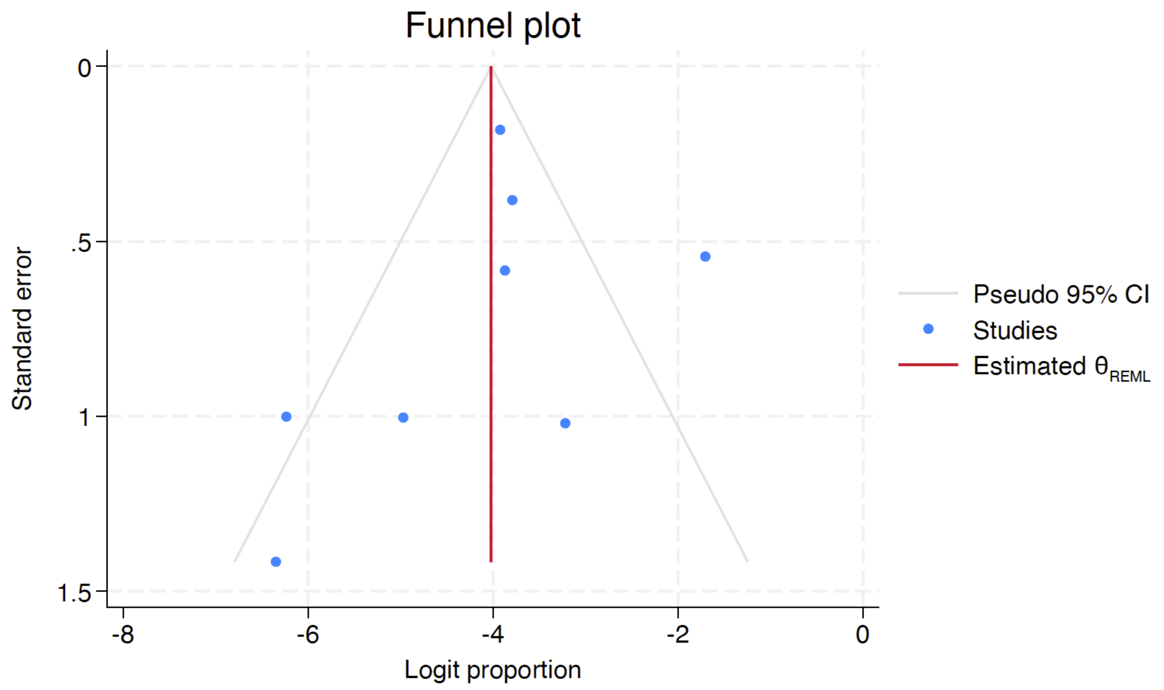

Figure S16. Meta-regression analysis of pericardial involvement as per study size

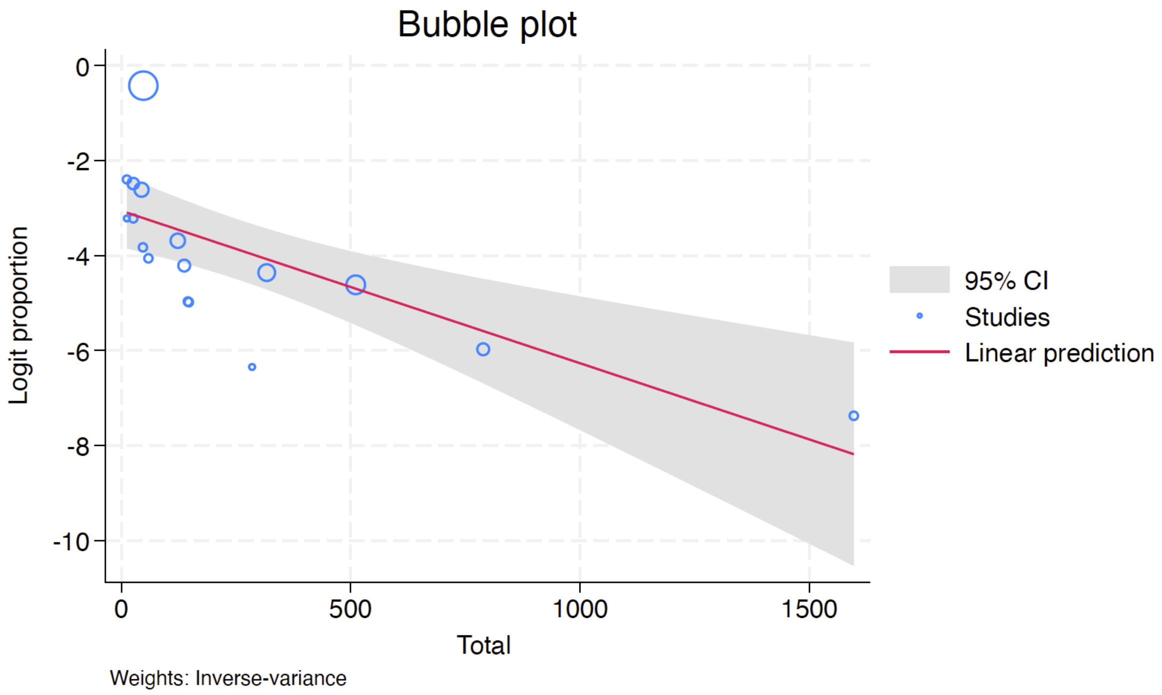

Figure S17. Sensitivity analysis for pericardial involvement including only large studies (with >200 participants)

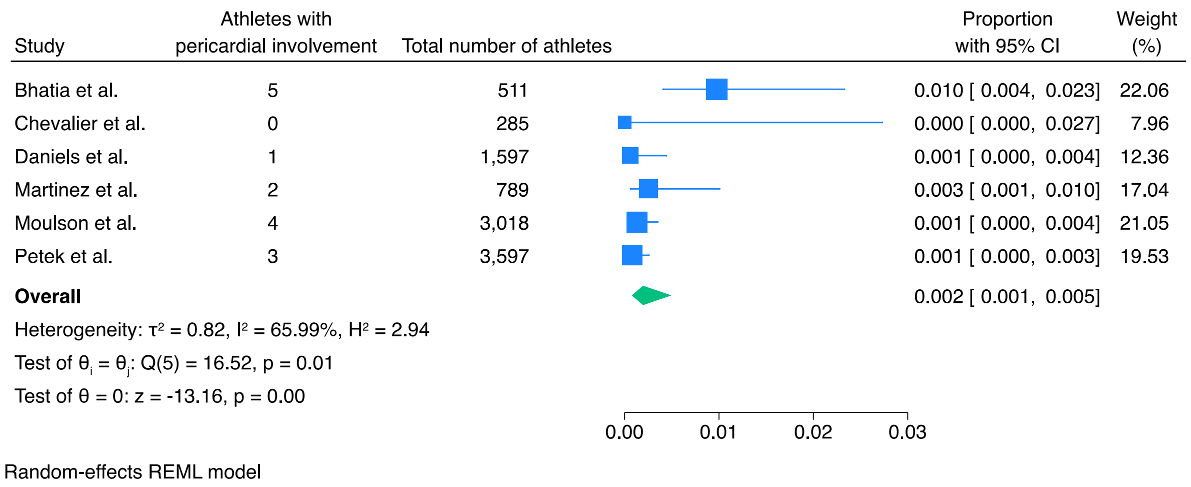

Figure S18. Funnel plot of pericardial involvement demonstrating significant small-study effects

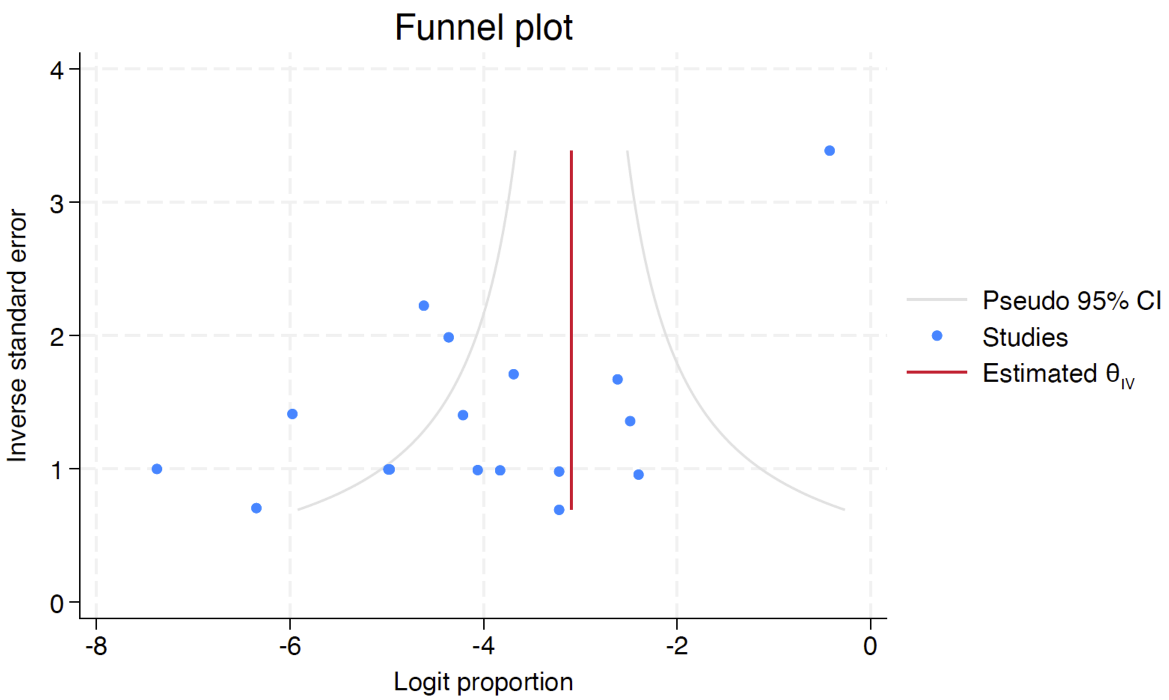

Figure S19. Funnel plot of pericardial involvement showing no significant publication bias

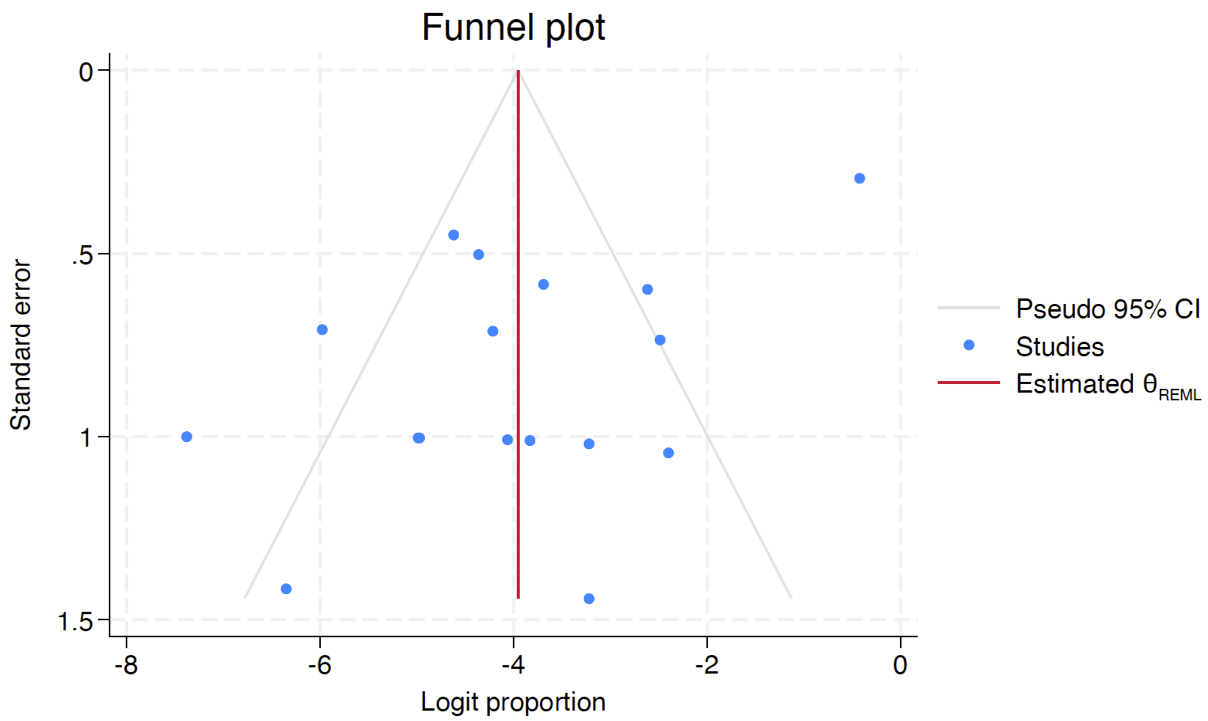

Figure S20. Sensitivity analysis for pericardial enhancement including the studies in which CMR was performed when clinically indicated (presence of symptoms and / or abnormal initial screening

Study	Athletes with pericardial enhancement	Total number of athletes	Proportion with 95% CI	Weight (%)
Bhatia et al.	5	17	0.294 [0.128, 0.542]	17.95
Brito et al.	19	48	0.396 [0.269, 0.539]	19.17
Chevalier et al.	0	102	0.000 [0.000, 0.073]	11.50
Hendrickson et al.	2	137	0.015 [0.004, 0.056]	16.73
Martinez et al.	2	27	0.074 [0.019, 0.252]	16.57
Moulson et al.	4	119	0.034 [0.013, 0.086]	18.09
**Overall**			0.070 [0.017, 0.245]	

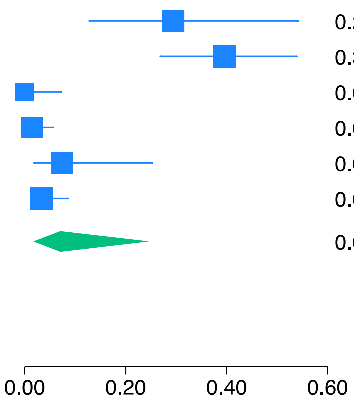

Heterogeneity: $\tau^2 = 2.80$, $I^2 = 89.95\%$, $H^2 = 9.95$

Test of $\theta_i = \theta_j$: $Q(5) = 51.63$, $p = 0.00$

Test of $\theta = 0$: $z = -3.47$, $p = 0.00$

Random-effects REML model
